# Prediction of Drug Combinations by Integrating Molecular and Pharmacological Data

**DOI:** 10.1371/journal.pcbi.1002323

**Published:** 2011-12-29

**Authors:** Xing-Ming Zhao, Murat Iskar, Georg Zeller, Michael Kuhn, Vera van Noort, Peer Bork

**Affiliations:** 1 European Molecular Biology Laboratory (EMBL), Heidelberg, Germany; 2 Biotechnology Center, Technical University Dresden, Dresden, Germany; 3 Max-Delbrück-Centre for Molecular Medicine, Berlin, Germany; Stanford University, United States of America

## Abstract

Combinatorial therapy is a promising strategy for combating complex disorders due to improved efficacy and reduced side effects. However, screening new drug combinations exhaustively is impractical considering all possible combinations between drugs. Here, we present a novel computational approach to predict drug combinations by integrating molecular and pharmacological data. Specifically, drugs are represented by a set of their properties, such as their targets or indications. By integrating several of these features, we show that feature patterns enriched in approved drug combinations are not only predictive for new drug combinations but also provide insights into mechanisms underlying combinatorial therapy. Further analysis confirmed that among our top ranked predictions of effective combinations, 69% are supported by literature, while the others represent novel potential drug combinations. We believe that our proposed approach can help to limit the search space of drug combinations and provide a new way to effectively utilize existing drugs for new purposes.

## Author Summary

The combination of distinct drugs in combinatorial therapy can help to improve therapeutic efficacy by overcoming the redundancy and robustness of pathogenic processes, or by lowering the risk of side effects. However, identification of effective drug combinations is cumbersome, considering the possible search space with respect to the large number of drugs that could potentially be combined. In this work, we explore various molecular and pharmacological features of drugs, and show that by utilizing combinations of such features it is possible to predict new drug combinations. Benchmarking the approach using approved drug combinations demonstrates that these feature combinations are indeed predictive and can propose promising new drug combinations. In addition, the enriched feature patterns provide insights into the mechanisms underlying drug combinations. For example, they suggest that if two drugs share targets or therapeutic effects, they can be independently combined with a third common drug. The ability to efficiently predict drug combinations should facilitate the development of more efficient drug therapies for a broader range of indications including hard-to-treat complex diseases.

## Introduction

In the past decades, targeted therapies modulating specific targets were considerably successful. However, recently, the rate of new drug approvals is slowing down despite increasing research budgets for drug discovery. One reason for this is that most human diseases are caused by complex biological processes that are redundant and robust to drug perturbations of a single molecular target. Therefore, the ‘one-drug-one-gene’ approach is unlikely to treat these diseases effectively [1].

Drug combinations can potentially overcome these limitations: they consist of multiple agents, each of which has generally been used as a single effective drug in clinic. Since the agents in drug combinations can modulate the activity of distinct proteins, drug combinations can help to improve therapeutic efficacy by overcoming the redundancy underlying pathogenic processes. In addition, some drug combinations were found to be more selective compared to single agents [2], thereby reducing toxicity and side effects. Nowadays, drug combinatorial therapy is becoming a promising strategy for multifactorial complex diseases. For example, thiazide diuretics cause hypokalaemia when used to treat hypertension, while this side effect can be prevented by angiotensin-converting enzyme (ACE) inhibitors when they are used concurrently [3]. Saracatinib can overcome the resistance of breast cancer to trastuzumab when both drugs are used together, thereby improving the efficacy of trastuzumab [4]. Both glyburide and metformin are indicated for type 2 diabetes but work in different ways: glyburide reduces insulin resistance while metformin increases insulin secretion, and therefore the combination of these two drugs can improve therapeutic efficacy due to their complementary mechanisms [5].

Despite the increasing number of drug combinations in use, many of them were found in the clinic by experience and were not designed as such; the molecular mechanisms underlying these drug combinations are often not clear, which makes it difficult to propose new drug combinations. High-throughput screening was found to be useful to identify possible drug combinations [6]; however, it is impractical to screen all possible drug combinations for all possible indications since it leads to an exponential explosion as the number of drugs increases. Therefore, similarly to drug-target predictions [7], [8], [9], [10], a number of computational methods for predicting drug combinations have recently been developed. For example, stochastic search techniques were used to identify optimal combinations within a large parameter space [11] in an iterative way, but they only work on small drug sets due to the computational and experimental cost. Mathematical modeling was used to determine synergistic combinations by comparing dose-response profiles of single agents against those of drug combinations [12], but it cannot explain the molecular mechanisms that underlie the drug combinations. Recently, in systems biology, both quantitative [13] and qualitative [14] models were introduced to investigate drug combinations based on the molecular networks or pathways possibly affected by the drugs. Although network analysis, in principle, can provide insights into the molecular mechanisms of drug actions [15], the incompleteness of molecular networks and the scarceness of the corresponding kinetic parameters limit the application of such approaches to drug combinations considerably.

In general, drugs are combined based on their mechanisms of action, which is characterized by the properties of drugs, such as their targets and pharmacology [16], [17]. Taking this into account, we present here a novel concept for the prediction of drug combinations that integrates both molecular and pharmacological features associated with drugs. We treated drug combinations as combinations of their corresponding features, including their target proteins, therapeutic effects, and indication areas. Analysis on the drug combinations approved by the US Food and Drug Administration (FDA) demonstrates that there are some feature patterns enriched in known combinatorial therapies that are both predictive of new drug combinations and provide insights into the mechanisms underlying combinatorial therapy. We consequently predict new drug combinations based on feature patterns enriched in approved drug combinations. Subsequent targeted literature survey revealed that 69% of our predictions were previously reported as effective combinations although they are not approved yet, corroborating the predictive power of our proposed method. In addition, we identify several novel potential drug combinations. For example, we predict a novel combination of promethazine and ibuprofen that could be used as decongestant. Although experimental validation of each individual prediction needs to be provided in the future, we believe that our proposed approach can guide the selection of drug combinations to be tested experimentally.

## Results/Discussion

### Drug features of approved drug combinations

**Figure 1:**
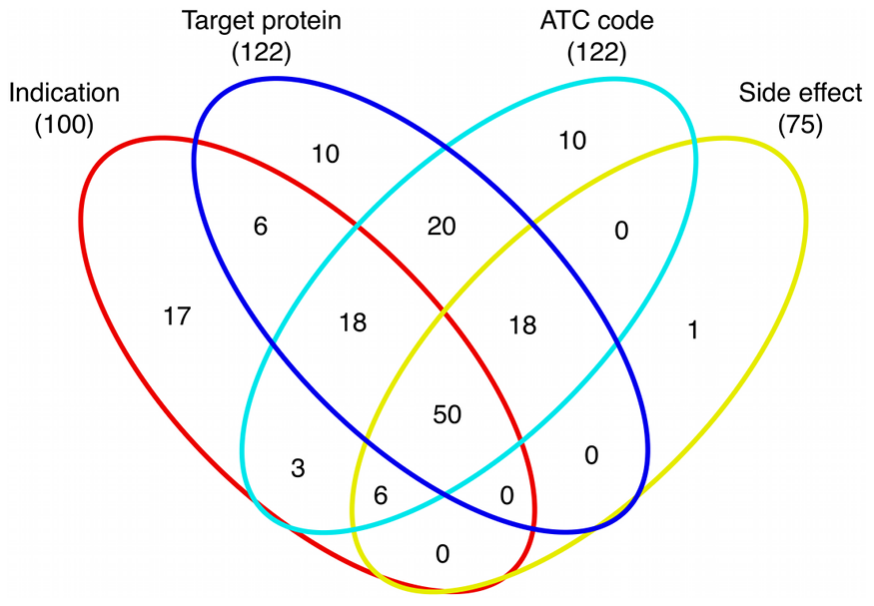
The Venn diagram of drug combinations, where the numbers indicate how many drug combinations can be covered by available features.

In order to predict potential drug combinations, we first identified properties of approved pairwise drug combinations. A total of 184 pairwise drug combinations (involving 238 drugs) were approved by the FDA until November 2010 (see Table S1). We collected the molecular and pharmacological information associated with these drugs, including their target proteins and corresponding downstream pathways, medical indication areas, therapeutic effects as represented in the Anatomical Therapeutic Chemical (ATC) Classification System, and side effects. Here, each such property of a drug is called a feature, and a feature pair means two feature variables respectively associated with two different drugs. Therefore, a drug pair can be represented as a vector composed of feature pairs. For example, in case of the feature ‘target protein’, drug 1 binds two proteins {p1, p2}, drug 2 binds three proteins {p3, p4, p5}, the combination of drug 1 and drug 2 can be represented as following feature pairs: {(p1, p3), (p1, p4), (p1, p5), (p2, p3), (p2, p4), (p2, p5)}, and similarly for other features. The numbers of drug combinations with available features are shown in Figure 1 (drug combinations with pathway annotations are not shown because they are a subset of those with target annotations).

### Target protein pairs are repeatedly used in drug combinations

**Figure 2:**
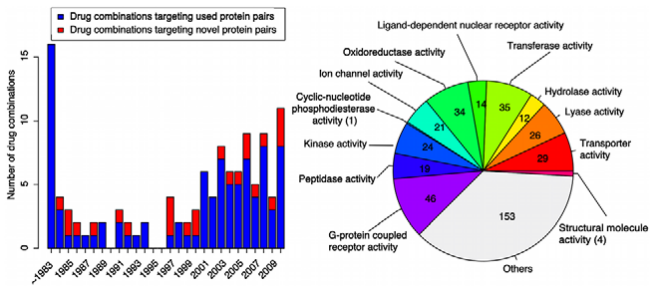
Historical distribution of drug combinations with respect to novel target combinations. (a) Distribution of pairwise drug combinations that target novel protein pairs or ones that are also targeted by previous combinations for the time between 1984 and 2010. (b) Distribution of drug targets with respect to molecular functions from Gene Ontology.

Focusing on the ‘target protein’ feature, we investigated the drug combinations approved each year between 1984 and 2010 and observed that protein pairs targeted by newly approved combinations have often already been the targets of previous drug combinations. In total, 117 drug pairs approved as effective combinations until November 2010 have specific targets, when metabolizing enzymes and unspecific protein binders are excluded [16]. With the 16 drug combinations approved until 1983 as baseline, we found that as many as 76% (77/101) of drug combinations approved during 1984 and 2010 are directed against 6039 unique protein pairs (418 proteins) that had been targeted previously by other combinations (Figure 2 (a)). According to annotations from Gene Ontology [18], we investigated the molecular functions of these 418 repeatedly used proteins (Figure 2 (b)), and found that these target proteins cover a broad range of functions, which implies that drug combinations are not biased towards specific classes of protein targets. The above observations indicate that there are some target protein patterns enriched in previously approved drug combinations, which can be used to predict new drug combinations.

### Benchmarking enriched molecular and pharmacological feature patterns to predict drug combinations

Encouraged by the enrichment of certain protein patterns in approved drug combinations, we investigated the possibility to use the five drug features described above for predicting drug combinations. For this, each feature pair was assigned a score by comparing its frequency in effective drug combinations with that in the background (see Materials and Methods, Eq. 1), which consists of all possible pairs of drugs involved in effective combinations. The detailed scores for each feature pair can be found in Tables S2, S3, S4.

**Figure 3:**
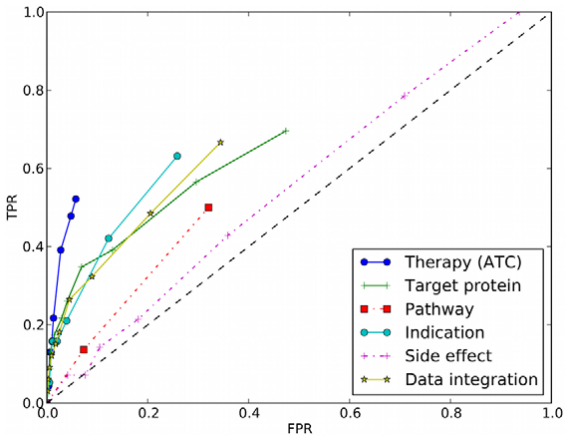
Performance of molecular and pharmacological features in 5-fold cross-validation, where the diagonal dashed line denotes random prediction.

To evaluate the predictive ability of these features, we performed 5-fold cross-validation on the 184 drug combinations extracted from FDA orange book [19] (see Materials and Methods). Figure 3 shows the receiver operating characteristics (ROC) curves obtained for different features, where the 5-fold cross-validation was repeated 10 times and the average was used as the final result (detailed results can be found in Table S5). Note that the performance of our method may be underestimated here because there are no true negative samples that are verified as invalid combinations. Furthermore, Figure 3 shows that all the features perform better than a random predictor, which implies that these properties can indeed help to predict new drug combinations. Among the features, the pathway feature was only weakly predictive, maybe because the simple association between drugs and pathways through target proteins does not sufficiently reflect the physiological context in which drugs work. One possible explanation for the observed poor predictive ability of the side-effect feature is that there are some common side effects associated with most drugs, thereby introducing much background noise. The performance of side effects may be improved if we consider only severe side effects associated with drugs. However, currently information on side-effect severity is unfortunately rarely available. The therapy information denoted by ATC code was found to be most predictive, probably due to the pharmacological information captured in the ATC code.

Given the incomplete coverage of drug combinations by different features (cf. Figure 1), we integrated the three most predictive features (i.e. therapy, target and indication area) for predicting new combinations hereinafter (see Materials and Methods, Eq. 3). A correlation analysis of feature similarities shows that some correlations between features exist (Figure S1). However, distinct data sources complement each other in the prediction of drug combinations since the coverage of each feature is incomplete and the overlap between different features is low as shown in Figure 1. By aggregating the three features where available, we aimed at improving the coverage of drug combinations compared with single features. For example, in 5-fold cross-validation, there are about 37 drug combinations in the validation set, among which 24 have ATC annotations while data integration by aggregating the three features can cover 34 drug combinations. In addition, the threshold above which data integration achieves the highest *F1* score in cross-validation was used to make future predictions (see Materials and Methods for details). Hereinafter, we set the threshold to 0.4, corresponding to an *F1* score of 0.17. If a new drug pair has an integration score above this threshold, it will be treated as a putative combination. Note that here we choose to use a simple method (maximization of the *F1* score) to predict whether a drug pair is an effective combination instead of other classifiers (e.g. support vector machine or Bayesian classifier). The advantage of this method is that it is easy to interpret and avoids overfitting when dealing with small sample sizes and an imbalance between positive and negative examples (i.e. all possible drug pairs except approved combinations) in our datasets.

### Predicting novel drug combinations

By aggregating the three features that have been shown to be most informative above, we then predict possible effective combinations between marketed drugs. For this, pairwise drug combinations from the FDA orange book were used as training set to assign an enrichment score for each feature pair, and the integration of these features was used to screen all possible combinations between drugs involved in known combinations. To identify novel combinations, we excluded pairs already known to be valid combinations. In our dataset, we found that the mechanisms of drug combinations indicated for hypertension and contraception are relatively well studied. The drugs involved in combinations for hypertension mainly include thiazide diuretics, ACE inhibitors, angiotensin II antagonists, and beta-blocking agents, while the majority of available combinations are diuretic-based [20]. In the case of drug combinations for contraception, estrogen is mainly combined with hormonal contraceptives or progestogens. Therefore, the drugs involved in these two kinds of combinations were not considered here while making new predictions.

As a result, we predict 16 possible drug combinations with confidence scores above the threshold of 0.4 (see Benchmarking) (Table 1); the detailed feature patterns and their corresponding scores involved in predictions can be found in Table S6. A literature survey showed that 11 out of our 16 predictions have already been reported to be effective in the literature (Table 1) although they have not yet been approved by the FDA. For example, metformin and glimepiride are being explored as a combinatorial treatment for type 2 diabetes with different but complementary mechanisms, and have shown promising results [21], [22], [23]. Some of our 11 predictions are also supported by other sources beyond the scientific literature. For example, ciprofloxacin and loteprednol etabonate have been patented as an effective combinatorial treatment (United States Patent 6359016). In summary, the large overlap (69%) between our predictions and those reported demonstrates that our proposed method effectively predicts new potential drug combinations.

**Table 1:** Predicted drug combinations and corresponding confidence scores, and PubMed IDs if they were reported in literature.

Agent 1	Agent 2	Confidence score	PubMed ID if confirmed in literature (included in combinations with more than 2 agents)
metformin hydrochloride	glimepiride	0.45	PMID: 11678974, PMID: 18849173, PMID: 16406190
niacin	atorvastatin calcium	0.45	PMID: 10095800
ibuprofen	pseudoephedrine sulfate	0.45	PMID: 15562884 (ibuprofen/pseudoephedrine/chlorpheniramine)
metformin hydrochloride	telmisartan	0.45	PMID: 20415664
promethazine hydrochloride	ibuprofen	0.45	
budesonide	ciprofloxacin	0.45	
loteprednol etabonate	ciprofloxacin	0.45	US Patent 6359016
fluoxetine hydrochloride	perphenazine	0.44	PMID:8104930
acetaminophen	morphine sulfate	0.44	PMID: 9706932
acetaminophen	buprenorphine	0.44	PMID: 7041936
ciprofloxacin	diclofenac sodium	0.44	PMID: 19301941
amitriptyline hydrochloride	olanzapine	0.44	PMID: 18172909
niacin	ezetimibe	0.44	PMID: 20152243 (ezetimibe/simvastatin/niacin)
methocarbamol	dipyridamole	0.44	
carisoprodol	dipyridamole	0.44	
ciprofloxacin	fluticasone propionate	0.44	

For the remaining 5 combinations, no literature support was found, implying that they are novel potential combination therapies. For example, we propose the combination of promethazine hydrochloride and ibuprofen based on the target combination (HRH3 and ALOX12) to relieve nasal blockage. In our training dataset, ibuprofen is combined with three drugs, i.e. diphenhydramine, phenylephrine, and pseudoephedrine. However, promethazine share neither chemical similarity nor therapeutic effects with any of these three drugs. Promethazine is known as histamine receptor H1 antagonist and used as an anesthetic agent. Ibuprofen is a nonsteroidal anti-inflammatory drug (NSAID) used for relief of symptoms of arthritis and pain. These two drugs are predicted to be an effective combination mainly based on the inhibition of histamine receptor H3 (HRH3) and arachidonate 12-lipoxygenase (ALOX12) by promethazine and ibuprofen respectively. Promethazine inhibits histamine that in turn increases human airway epithelial paracellular permeability [24], while 12-lipoxygenase deficiency was found to protect mice from allergic airway inflammation [25]. Based on their target information, we thus propose that promethazine and ibuprofen can be combined as decongestant.

Furthermore, ciprofloxacin and budesonide were predicted to be combinable because of the therapeutic effect combination between anti-inflammatory agents (coded as A07E) and antibacterials (coded by J01M). Ciprofloxacin is a synthetic antibiotic inhibiting DNA gyrase that is necessary to separate bacterial DNA, thereby blocking synthesis of bacterial DNA. Budesonide is an anti-inflammatory glucocorticoid steroid and is used to treat asthma. Recently, the composition of microbiota from the bronchial epithelium was found to be associated with asthma pathogenesis [26]. Therefore, a therapy that combines anti-inflammatory agents (such as budenoside) and antibacterials (such as ciprofloxacin) appears promising for treating asthma. In addition, fluticasone propionate was also predicted to be combinable with ciprofloxacin based on the therapeutic effect combination between corticosteroids (coded by D07A) and antibacterials (coded by J01M). Fluticasone propionate is a synthetic corticosteroid and is indicated for asthma and allergic rhinitis. Based on their respective therapeutic information, the combination of these two drugs appears promising for the treatment of asthma.

### Characteristics of feature patterns enriched in approved drug combinations

To gain more insights into the mechanisms of drug combinations, we investigated the enriched protein and therapy patterns that contribute to the integration score above the threshold of 0.4 as described above. For this, we constructed two drug-feature networks: a drug-protein network and a drug-therapy network for drug combinations that contain these enriched patterns. In these networks, two drugs were linked if they were documented as an effective combination and each drug was additionally linked to its features.

**Figure 4:**
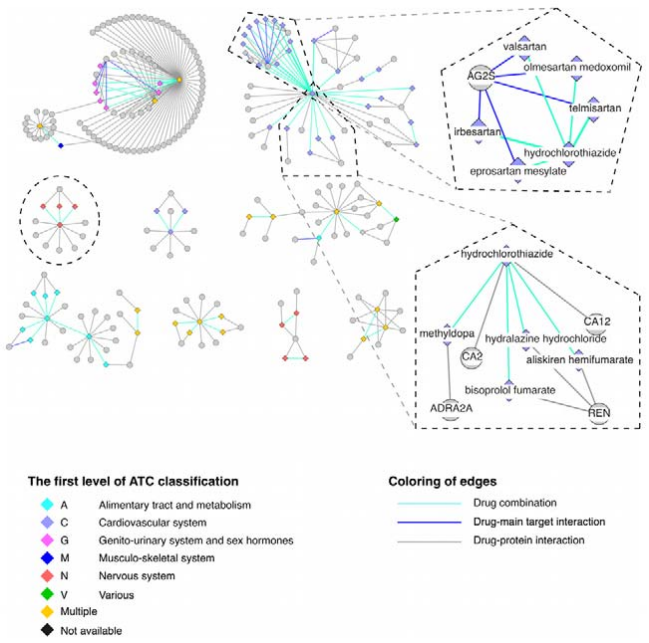
Drug combination and drug-protein network. A drug is linked to its target protein(s) and additionally to other drugs with which it can be combined. Only the protein pair(s) with the highest score for each drug pair is (are) included for clarity. Drugs are depicted as diamonds, proteins as circles. The color of drug nodes indicates its therapeutic category (the first level of ATC code), and drugs are labeled as multiple if they are associated with more than one ATC code. Cases discussed in the text are highlighted within dashed lines.

The drug-protein network (Figure 4) involving 59 drug combinations that contain enriched protein pairs shows that most of the drug pairs in the same combinations belong to the same general therapeutic category (the first level of the ATC code), and the detailed network can be found in Dataset S1. For two drugs that are found in approved combinations with a common third drug, we observed that they tend to share target proteins. Among 281 such pairs of drugs, 100 share target proteins - a significantly higher proportion than expected by chance (p-value of 10^−5^, Fisher's exact test). This phenomenon is more obvious for drugs sharing main targets. For example, five angiotensin II receptor antagonists - irbesartan, olmesartan medoxomil, valsartan, eprosartan mesylate and telmisartan - have type 1 angiotensin II receptor (AG2S) as main target, and all these five drugs can be combined with hydrochlorothiazide, a thiazide agent. This observation is not surprising since drugs that share the same target protein generally have similar pharmacology, thereby tending to be interchangeable with each other when combined with another drug for similar purposes. Furthermore, we ruled out the possibility that our identified protein patterns are a trivial consequence of high chemical similarity between drugs, which would in turn imply increased likelihood of targeting the same proteins [8] as well as similar pharmacology. By investigating the pairs of drugs in approved combinations with a third common drug, we found that only few of them have similar chemical structures. Among the 281 drug pairs, only 14 have chemical similarity larger than 0.6, indicating that our approach captures much richer descriptions of drug combinations than chemical structure similarity alone.

Furthermore, the feature patterns we identified here can help to explain the mechanisms of action of drug combinations. For example, hydrochlorothiazide, a diuretic drug, and methyldopa, an alpha-adrenergic agonist, are combined for the treatment of hypertension. Hydrochlorothiazide is very commonly combined with other drugs (Figure 4) for lowering blood pressure by reducing the kidney's ability to retain water, thereby resulting in reduced blood volume. At the molecular level, hydrochlorothiazide inhibits carbonic anhydrase 2 (CA2), a member of an enzyme family that catalyzes the release of water molecules from carbonic acid. Methyldopa is an agonist of alpha-2 adrenergic receptors (ADRA2A) that mediates the sympathetic nerve activity, which in turn leads to reduced renin activity and lower blood pressure [27]. With knowledge about the physiological roles of drug targets, protein feature pairs are indeed helpful for explaining the mechanism underlying the combination therapies.

**Figure 5:**
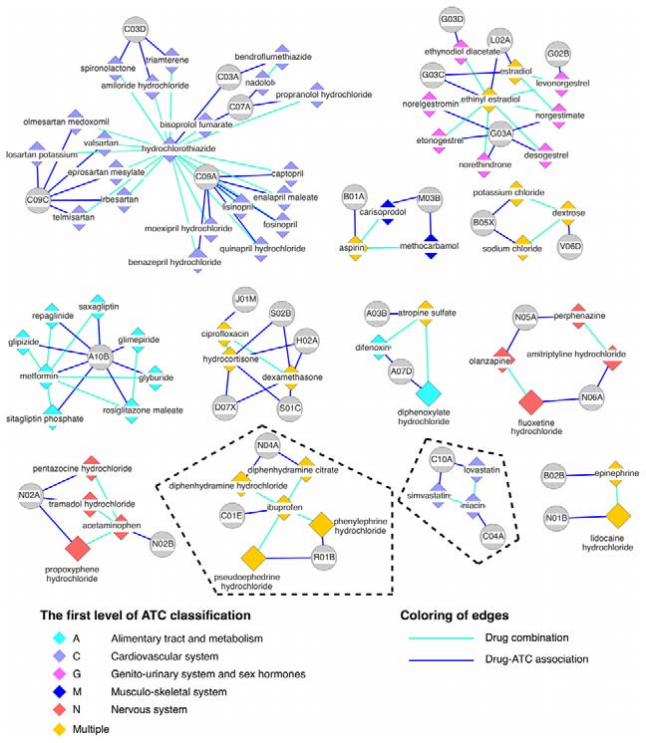
Drug combination and drug-therapy network. A drug is linked to its therapy, additionally drugs are linked if they can be combined, where the third level of the ATC code was considered because other levels are either too general to reveal pharmacological differences or too specific with too few common annotations left. Drugs are depicted by diamonds, therapies (according to ATC) as circles, and the color of each drug node denotes its therapeutic category (the first level of ATC code). Cases detailed in the text are highlighted within dashed lines.

The drug-therapy network (Figure 5) constructed for 55 drug combinations containing enriched therapy patterns (third level of the ATC code) reveals that drugs in combinations do not necessarily have therapeutic effects in common (the detailed network can be found in Dataset S2). In fact, only 9 out of 55 drug combinations share therapeutic effects, indicating that the agents in the same combination tend to complement each other with respect to their specific therapeutic effects although they belong to the same general therapeutic category. Furthermore, we found that two drugs that are in approved combinations with a common third drug tend to have similar therapeutic effects. Among the drugs shown in Figure 5, there are 205 such drug pairs, 77 of which share therapeutic effects indicating a significant enrichment (p-value<10^−6^, Fisher's exact test). For example, both lovastatin and simvastin can be combined with niacin for the treatment of dyslipidemia, where lovastatin and simvastin are peripheral vasodilators (ATC code C04A) and niacin is a lipid-modifying agent (ATC code C10A).

Analysis of the two drug-feature networks shown above demonstrates that our identified drug feature patterns can indeed provide insights into the mechanisms of action that underlie drug combinations.

### Conclusions

Our approach to predict drug combinations by representing drug combinations as combinations of their molecular and pharmacological features, including target proteins, therapies, and indication areas, not only led to the proposal of new drug combinations but also allowed mechanistic insights into existing ones. The overlap between our predictions and those reported in the literature demonstrate that this approach can effectively identify new drug combinations with the enriched feature patterns as an indicator for the mode of action underlying both marketed and predicted drug combinations. A limitation of this method is that it relies on the feature patterns enriched in approved drug combinations, which limits our predictions to those combinations that are similar to existing ones to some extent. Nevertheless, the new combinations are far from being obvious given the vast space of possible solutions. We believe that the methods proposed here can limit the search space of possible drug combinations as a guide for experimental screens and provide an alternative starting point towards repurposing old drugs.

## Materials and Methods

### Drug combinations, drug targets, drug therapy, drug indications, and drug side effects

All drug combinations were parsed from the FDA orange book [19] (up to November, 2010), and only pairwise combinations of prescription and over-the-counter (OTC) drugs were considered here. In total, our data set contains 184 drug combinations and 238 drugs.

For drug target annotations, we used the compound-protein interactions from STITCH (version 2) database [28], requiring a confidence score higher than 0.7 and supported by either database or experiments. Furthermore, we combined this information with data collected from DrugBank (version 3) [29] and therapeutic target database (TTD, November, 2010) [30]. In particular, the targets from the TTD database were treated as main targets because they are annotated as primary therapeutic targets of drugs. We further investigated the pathways possibly affected by a drug through its target(s), where pathway information was retrieved from the KEGG database [31]. For drug-pathway associations, each drug was associated with the pathways in which its target proteins are found.

Drug indications were extracted from drug package inserts. Due to different names and synonyms associated with a disease, we mapped all disease names to Medical Subject Headings (MeSH) [32] terms by exact match, considering only the diseases branch and the psychiatry and psychology branch. Drug side effect information was retrieved from the SIDER database [33]. Drug therapy information was extracted from both STITCH and DrugBank, where the therapy information is represented as Anatomical Therapeutic Chemical (ATC) Classification System. Specifically, the third level of the ATC code was used here to represent the therapy information for each drug. Chemical similarity was calculated as the two-dimensional Tanimoto chemical similarity score with the Chemistry Development Kit [34].

The drug-protein network was constructed for drug combinations that contain enriched protein patterns, where two drugs were linked if they are an effective combination and each drug was also linked to its targets, and the same for the drug-therapy network. The networks were visualized with Cytoscape [35].

### Prediction of drug combinations

For each drug, the information extracted above can be used to describe the drug, including targets, indications, pathways, therapies encoded by ATC code, and side effects. For a drug pair 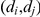 and a feature *F* (e.g. drug target), 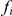 is associated with 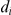 and 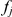 is associated with 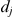, where 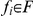 and 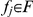. Therefore, drug pair 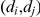 can be represented as feature pair 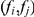. For each feature pair 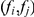, a score 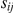 is calculated as follows.
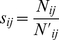
(1)where 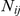 is the number of times that feature pair 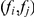 occurs in effective drug combinations, and 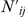 is the number of times that feature pair 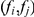 occurs in the background set of all possible pairwise combinations between drugs involved in known drug combinations. In this way, all the feature pairs can be ranked based on their scores and those ranked top are the feature pairs most strongly enriched in drug combinations.

After getting the feature pairs for each drug combination, we used 5-fold cross-validation to evaluate their performance. In the 5-fold cross-validation, all the drug combinations were randomly split into five groups with similar size without overlap, four of which were used as training set and used to calculate the enrichment score for each feature pair while the remaining group was used as the validation set to evaluate the performance of the feature pairs, and the procedure was repeated for five times. The *F1* score defined below was adopted as performance index.
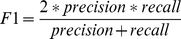
(2)where precision is the ratio of true positives in predicted positives and recall is the ratio of true positives that can be predicted correctly. The threshold above which the highest *F1* score was achieved in cross-validation was used to make future prediction. We predict a drug pair as an effective combination if its score is above the threshold.

Since the annotations from different data sources are incomplete, the feature pairs from distinct data sources were aggregated to calculate a confidence score about whether two drugs can be combined with the hope that information from distinct data sources can complement each other. For a drug pair 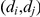, the confidence score is defined as follows.
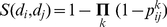
(3)where 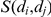 is the confidence or probability of drug 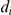 combining with drug 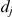, 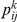 is the confidence that drug 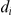 can be combined with drug 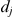 based on feature pairs from data source *k* (e.g. target), and 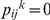 if the drug pair have no corresponding information from data source *k*. The feature confidence 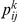 is defined as follows.
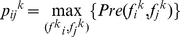
(4)where 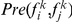 is the precision obtained with feature pairs whose scores (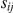) are larger than that of feature pair 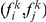, and the maximum is used because there are possibly multiple feature pairs for one drug pair from data source 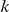.

## Supporting Information

Figure S1 **Correlation analysis between different features.** For each feature, e.g. target protein, one vector with dimensionality of *m* (i.e. the total number of approved drug combinations) is constructed, where each element denotes the highest score achieved by the feature pairs associated with the corresponding drug pair based on Eq.1. Subsequently, the spearman correlation coefficient is calculated between different features.
(TIF)

Dataset S1 **The drug-protein network in Cytoscape format.** In the network, the edge property of ‘dd’ denotes drug-drug associations that are approved combinations, and ‘dp’ drug-protein interactions while ‘d_m’ denotes the interactions between drugs and their main targets.
(TSV)

Dataset S2 **The drug-therapy network in Cytoscape format.** In the network, the edge property of ‘dd’ denotes drug-drug associations that are approved combinations and ‘da’ drug-therapy (represented as ATC code) associations.
(TSV)

Table S1 **All pairwise drug combinations parsed from FDA orange book.**
(XLSX)

Table S2 **Protein pairs with corresponding scores based on all known drug combinations.**
(XLSX)

Table S3 **Therapeutic effect (ATC code) pairs with corresponding scores based on all known drug combinations.**
(XLSX)

Table S4 **Disease (MeSH code) pairs with corresponding scores based on all known drug combinations.**
(XLSX)

Table S5 **5-fold cross-validation results obtained by different features.**
(XLSX)

Table S6 **Detailed features used for predicted drug combinations, where only the feature pattern with the highest score from each feature is shown for clarity.**
(XLSX)
