# Supplementary figures and images for: Prediction of Drug Combinations by Integrating Molecular and Pharmacological Data
Source: PLoS Comput Biol. 2011 Dec 29;7(12):e1002323. doi: 10.1371/journal.pcbi.1002323 (PMC3248384; doi:10.1371/journal.pcbi.1002323)

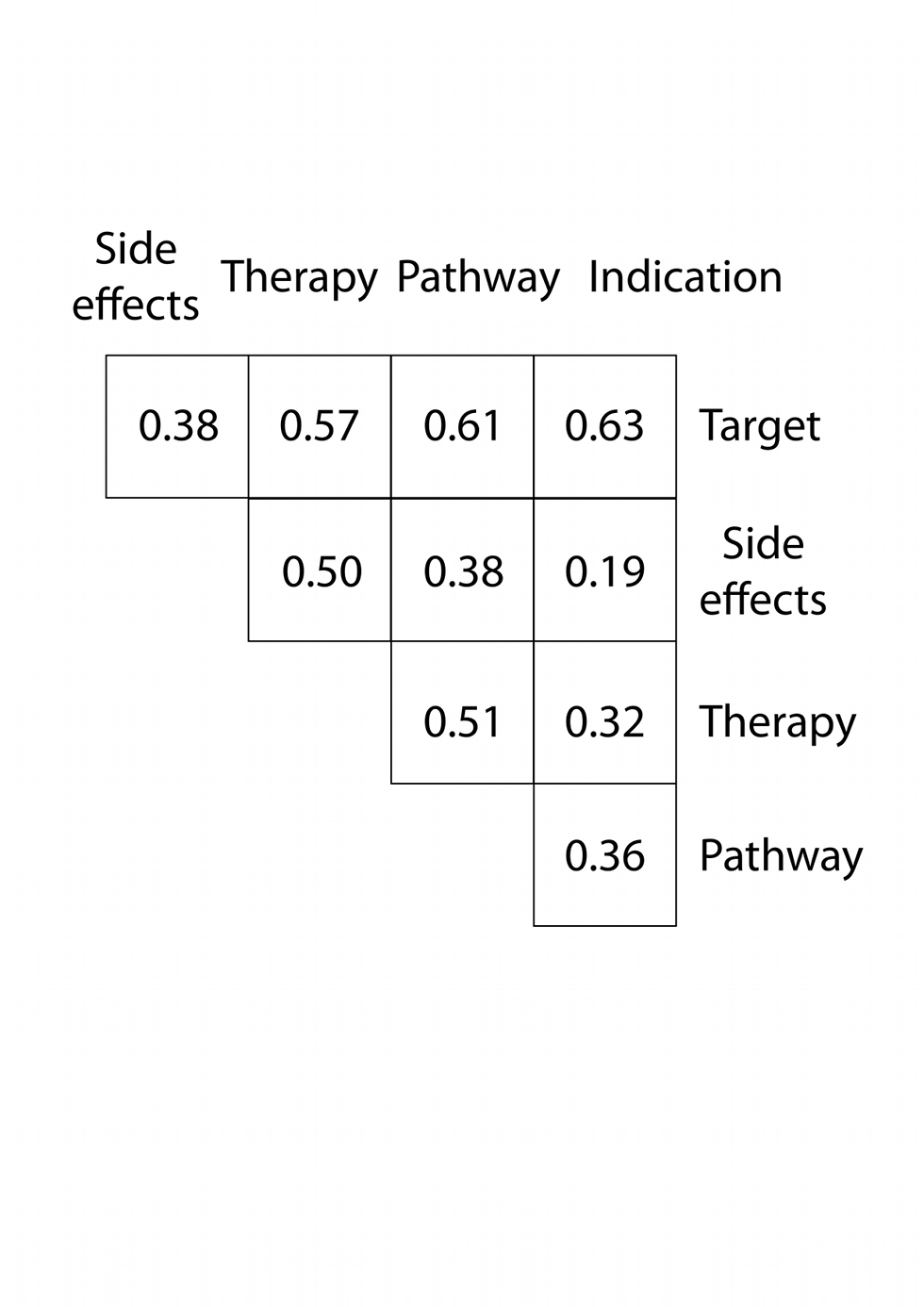

Supplement: Figure S1 — Correlation analysis between different features. For each feature, e.g. target protein, one vector with dimensionality of m (i.e. the total number of approved drug combinations) is constructed, where each element denotes the highest score achieved by the feature pairs associated with the corresponding drug pair based on Eq.1. Subsequently, the spearman correlation coefficient is calculated between different features. (TIF) [file pcbi.1002323.s001.tif]
